# Editorial: How to Help Employees Returning to Work Following Depression

**DOI:** 10.3389/fpsyt.2021.714589

**Published:** 2021-06-25

**Authors:** Takeshi Terao, Hikaru Hori, Claudia Buntrock

**Affiliations:** ^1^Department of Neuropsychiatry, Faculty of Medicine, Oita University, Yufu, Japan; ^2^Department of Psychiatry, Faculty of Medicine, Fukuoka University, Fukuoka, Japan; ^3^Depaatment of Psychiatry, University of Erlangen, Erlangen, Germany

**Keywords:** work restoration, work maintenace, re-work program, stigma, education, depression

Workers who are on sick leave due to common mental health disorders, like major depressive disorder, have difficulty in returning to work. Recurrent sickness absences are often more serious and long-lasting than the first sickness absence episode. Additionally, frequent sickness absences are related to an increased risk of work disability. Despite these circumstances, our knowledge of how to prevent recurrent sickness absence after return to work and of barriers to accessing effective interventions and work accommodations for mental health disorders is limited. Therefore, it is important to collect as much evidence as possible investigating which interventions improve occupational functioning when returning to work after common mental health disorders.

On this topic, we present six excellent papers, of which three Ohki et al., Hayasaka et al., and Hoaki and Terao investigated the effects of support programs for restoration to workplaces in Japan, two Hayasaka et al. and Katagiri et al. described a case report in Canada and case series in Japan, respectively, and one Dewa et al. showed workers' preferences for disclosing their mental health issues to their managers and the consequences in the Netherlands. Since four of the six studies were conducted in Japan, the findings might have been somewhat deviated to Japanese society. Nonetheless, we believe that the findings may be possibly applied to other countries with caution, and at least it is warranted to investigate them in other countries.

Fear of stigma and discrimination can inhibit workers from disclosing their mental health problems to their managers. Dewa et al. showed that workers have diverse preferences for disclosing their mental health problems to their managers. The study's findings suggest that dealing with stigma in the workplace is not as simple as requiring all workers to undergo anti-stigma training. Rather, workers should be supported to make the appropriate disclosure decision based on their workplace contexts. Dewa et al. emphasized the importance of workplace educational programs and interventions to address workplace stigma. Before or along with performing a support program for restoration, such programs and interventions are necessary, which may let mentally sick workers honestly disclose their mental problems to their managers with a certain reward of warm-hearted support from their workplaces.

There are various support programs for restoration. One of them is a re-work program Ohki et al. and Hoaki and Terao, which is classified into five types (individual program, specific psychology program, educational program, group program, and other programs such as exercise and personal interviews), but some of the five types are usually combined in individual facilities according to their policy and the ability of staffs. Ohki et al. showed that work maintenance after restoration was significantly longer in subjects receiving a re-work program plus treatment as usual than in subjects receiving treatment as usual alone, suggesting that a re-work program may be effective for work maintenance. Hoaki and Terao revealed that the frequency of participation in a re-work program was positively associated with work restoration but not with work maintenance, indicating that the frequency can predict successful work restoration but not work maintenance. Hayasaka et al. explored factors related to finding employment within 1-year after discharge in patients with mood disorder who participated in an inpatient employment support program, which aimed to assess cognitive function and work performance of patients. Findings disclosed that work speed and mood response in the support program were significant predictors for finding employment.

Wisenthal described a case report that offers a concrete example of how cognitive work hardening (CWH), a multi-element, occupationally-based intervention, designed to close the gap between improvements in depression symptoms and work functioning, can be utilized in clinical practice. The case report provides insight into how CWH can be applied to prepare for re-entry into the workforce after depression with positive results. Katagiri et al. showed the details of interpersonal psychotherapy, which can improve treatment-refractory depression, which is a barrier to work restoration. The case series suggests that the appropriate selection of interpersonal psychotherapy might possibly lead to positive outcomes, including work productivity, and employment status in patients with treatment-refractory depression.

One of the important parts of support programs for restoration is for individual participants to look back over the past days when they began to take sick leaves from jobs and to think about the causes of which may be sorted into their own problems and others' ones. Their own problems include various psychological ones such as personality with immature, selfish, or inflexible tendency as well as biological ones such as depression. In any case, it is expected that through the programs, they can start reflecting on their psychological problems and try to resolve them; however, it may take much longer time than the duration of participation in the programs. Therefore, although support programs for restoration are very useful for mentally sick workers to restore to their jobs, the workers should recognize them as starting points, and continue to reflect on themselves for fostering their minds to cooperate with other workers with a feeling of worthwhile jobs. This is their own long-standing psychotherapy together with psychiatric outpatient treatment.

## Author Contributions

All authors listed have made a substantial, direct and intellectual contribution to the work, and approved it for publication.

## Conflict of Interest

The authors declare that the research was conducted in the absence of any commercial or financial relationships that could be construed as a potential conflict of interest.

